# Ticagrelor Versus Clopidogrel in Patients with Acute Coronary Syndrome and Chronic Kidney Disease: A Real-World Analysis from a National Registry

**DOI:** 10.3390/medicina61101804

**Published:** 2025-10-08

**Authors:** Tzu-Lin Wang, Victor Chien-Chia Wu, Kou-Gi Shyu, I-Chang Hsieh, Tien-Hsing Chen, Ming-Lung Tsai

**Affiliations:** 1 Division of Cardiology, Department of Internal Medicine, Shin-Kong Wu Ho-Su Memorial Hospital, Taipei 111, Taiwan; 2 Division of Cardiology, Chang Gung Memorial Hospital, Linkou Medical Center, Taoyuan 333, Taiwan; 3 College of Medicine, Chang Gung University, Taoyuan 333, Taiwan; 4 Division of Cardiology, Department of Internal Medicine, Keelung Chang Gung Memorial Hospital, Keelung 204, Taiwan; 5 Division of Cardiology, Department of Internal Medicine, New Taipei Municipal TuCheng Hospital, New Taipei 236, Taiwan; 6 College of Management, Chang Gung University, Taoyuan 333, Taiwan

**Keywords:** ticagrelor, clopidogrel, acute coronary syndrome, chronic kidney disease, CV outcome

## Abstract

*Background and Objectives:* Dual antiplatelet therapy (DAPT) with aspirin and a P2Y12 inhibitor is standard care for acute coronary syndrome (ACS). Although ticagrelor showed superiority over clopidogrel in pivotal trials, patients with advanced chronic kidney disease (CKD) or on dialysis were underrepresented and results in Asian populations have been inconsistent. *Materials and Methods:* We conducted a retrospective cohort study using the Taiwan Society of Cardiology Acute Coronary Syndrome-Diabetes Mellitus (TSOC ACS-DM) registry between 1 October 2013, and 30 September 2016. Eligible patients had type 2 diabetes mellitus and ACS with stage III–V CKD or were on dialysis at index hospitalization and were discharged on aspirin plus either ticagrelor or clopidogrel. The primary endpoint was a composite of cardiovascular (CV) death, CV-related readmission, and repeated revascularization. Cumulative incidence functions were compared using expectation maximization (EM) weighting and propensity score adjustment. *Results*: After exclusions, 451 patients were analyzed (ticagrelor n = 116; clopidogrel n = 335). Ticagrelor associated with higher myocardial infarction (HR 1.59, 95% CI 1.12–2.28, *p* = 0.010), CV-related readmission (HR 1.72, 95% CI 1.12–2.65, *p* = 0.014), repeated revascularization (HR 2.24, 95% CI 1.36–3.68, *p* = 0.002), and the composite endpoint (HR 1.63, 95% CI 1.06–2.48, *p* = 0.024) at 2 years. *Conclusions*: Among real-world Taiwanese patients with type 2 diabetes mellitus, ACS, and CKD, ticagrelor use was linked to increased risks of cardiovascular events compared to clopidogrel. However, these relationships might be affected by potential confounding factors. Randomized controlled trials are necessary to establish the best antiplatelet strategy for this high-risk group.

## 1. Introduction

Dual antiplatelet therapy (DAPT) with aspirin and clopidogrel has been the mainstay of treatment for acute coronary syndrome (ACS) for the past decades [1]. However, the clinical efficacy of clopidogrel is limited by inter-individual variability in platelet inhibition, largely attributable to genetic polymorphisms such as CYP2C19 loss-of-function alleles [2,3]. To overcome these limitations, more potent P2Y12 inhibitors were developed and subsequently demonstrated superior ischemic protection compared with clopidogrel in pivotal clinical trials. Current international guidelines therefore recommend ticagrelor as the preferred P2Y12 inhibitor in patients with ACS [4,5,6]. In the landmark PLATO trial, ticagrelor was associated with a 16% relative risk reduction in major adverse cardiovascular events (MACE) and a 22% reduction in all-cause mortality compared with clopidogrel, albeit at the expense of increased bleeding and dyspnea [7,8].

Despite these benefits, the generalizability of PLATO results has been questioned. Canadian clinical trials and other analyses have reported that replacing clopidogrel with ticagrelor in the DAPT may, in fact, lead to non-decreased coronary events with increased bleeding risks [9,10]. Patients with ACS are frequently complicated with chronic kidney disease (CKD) that may affect the antiplatelet activities. Subgroup analyses suggested that ticagrelor reduced ischemic endpoints and mortality in patients with moderate CKD without significantly increasing major bleeding [11]. However, advanced CKD and dialysis patients were markedly underrepresented, leaving uncertainty regarding the balance of risks and benefits in this high-risk population. Moreover, observational studies and randomized trials conducted in East Asian populations, such as PHILO (Japan) and TICAKOREA (Korea), failed to confirm clear superiority of ticagrelor over clopidogrel, and in some cases showed higher bleeding risk [12,13]. Real-world studies from Taiwan and other Asian countries have further reported neutral or even unfavorable outcomes with ticagrelor, particularly among patients with CKD or undergoing dialysis [14,15,16].

Given the high prevalence of CKD among patients with ACS and the complex interactions between renal dysfunction, platelet biology, and antiplatelet drug response, there is an urgent need for region-specific evidence to guide optimal therapy. Considering these uncertainties, we conducted a large-scale retrospective cohort study using a nationwide registry to evaluate the comparative effectiveness of ticagrelor versus clopidogrel in Taiwanese patients with ACS and concomitant stage III–V CKD or dialysis dependence.

## 2. Methods

### 2.1. Data Source

In this retrospective registry study, patient data were obtained from Taiwan Society of Cardiology Acute Coronary Syndrome-Diabetes Mellitus Registry (TSOC ACS-DM). The registry includes patients with ST-segment elevation myocardial infarction (STEMI), non–ST-segment elevation myocardial infarction (NSTEMI), or unstable angina. From 2013 to 2016, the registry recruited 2357 adult patients (≥20 years of age) with ACS who received percutaneous coronary intervention (PCI) during hospitalization from 24 hospitals [17]. The cardiologists who recruited the cases were responsible for diagnosis and management, based on recommendations from international and local guidelines [5,18,19]. Patients have signed the informed consent for their information be collected in the registry. Ethics committee approval was obtained at each trial site.

### 2.2. Study Patients

By searching electronic medical records from the TSOC ACS-DM Registry data between 1 October 2013 and 30 September 2016, we retrieved ACS and type 2 diabetic patients with stage III-V CKD or on dialysis. We excluded patients with missing data, in-hospital death, no prescribed with clopidogrel or ticagrelor at discharge, or prescription of both clopidogrel and ticagrelor. We then separate patients into those taking ticagrelor and those taking clopidogrel as part of their DAPT post-ACS.

### 2.3. Covariate and Study Outcomes

Data including the patients’ baseline characteristics, medical history, risk factors, clinical diagnosis, medications at the time of admission and discharge, in-hospital lab data, revascularization interventions/procedures, and in-hospital and 1-year outcomes regarding mortality, recurrent nonfatal myocardial infarction (MI) and stroke were collected. Covariates included age, sex, body mass index, waist circumference, creatinine, estimated glomerular filtration rate (eGFR), CKD stage, diabetes mellitus duration, etiology of ACS, culprit lesion, thrombolysis in myocardial infarction (TIMI) flow, left main stenosis, number of vessels affected, culprit artery territory, left ventricular ejection fraction (LVEF), treatment during the admission, concomitant disease, history of event, lipid profile, smoking, glucose lowering drug, non-diabetes mellitus medication, and follow-up duration (Table 1).

Primary outcomes are death, cardiovascular (CV) death, myocardial infarction (MI) readmission, CV-related readmission, repeat revascularization (including percutaneous coronary intervention [PCI], coronary artery bypass grafting [CABG]), and composite outcome, including CV death, CV-related readmission, and repeated revascularization. Each patient was followed until the day of outcome occurrence, date of death or 31 December 2016, whichever came first. Outcomes were recorded based on discharge diagnoses made by treating physicians at each participating hospital. Data were collected by trained study coordinators and submitted to the central registry for verification.

### 2.4. Statistical Analysis

Patient characteristics between the two treatment groups were compared using Fisher’s exact test for categorical variables, independent sample t-test for continuous variables and Mann–Whitney U-test for skewed continuous variables (i.e., troponin I and NT-proBNP). In addition to inferential statistics, we also calculate the standardized difference (STD) between group where an absolute STD value less than 0.2 indicates a non-substantial difference. To reduce the potential confounding when comparing outcomes between the study groups, we used the expectation maximization (EM) and adjusted for propensity score to balance the difference between groups. The propensity score was estimated using a multivariable logistic regression model in which the study group was regressed on the selected covariates listed in Table 1 where the follow-up month was replaced with the index date. Propensity score matching (PSM) was not employed as the primary analysis, as it would have reduced the sample size and consequently diminished statistical power. Instead, the propensity score was incorporated as a covariate to control for confounding in the statistical analyses. PSM with 1:1 ratio was used only for primary composite outcome as a sensitivity analysis. A *p* value < 0.05 was considered to be statistically significant. Since this study was exploratory nature rather than a confirmatory trial, no adjustment of multiple testing (multiplicity) was applied in this study. All statistical analyses were performed using commercial software (SAS 9.4, SAS Institute, Cary, NC, USA).

## 3. Results

### 3.1. Study Population

**Figure 1:**
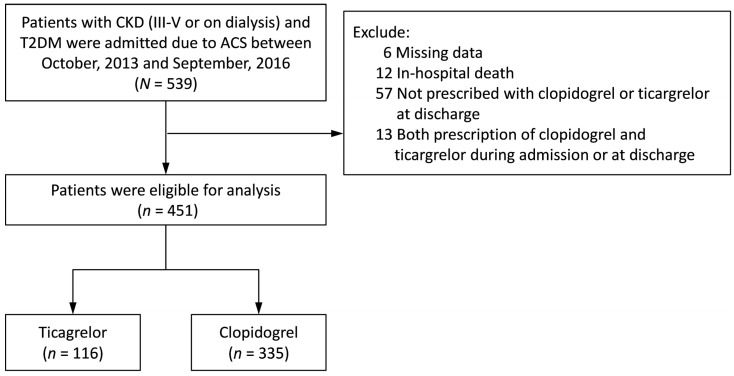
Study design and screening criteria flow chart for the inclusion of ACS patients.

There were 539 ACS and diabetic patients with stage III-V CKD or on dialysis between 2013 and 2016 identified from TSOC ACS-DM Registry. After excluding patients with miss data: 6, in-hospital death: 12, not prescribed with clopidogrel or ticagrelor at discharge: 57, and prescription with both clopidogrel and ticagrelor during admission or at discharge: 13. Finally, 451 patients were separated into 116 patients taking ticagrelor and 335 patients taking clopidogrel (Figure 1).

Patients taking ticagrelor had mean age 66.6 ± 11.2 with male 67.2% and patients taking clopidogrel had mean age 68.7 ± 10.5 with male 66.6% (Table 1). Patients taking ticagrelor had mean creatinine 3.0 ± 2.9, eGFR 36.9 ± 18.2, with 72.4% stage III, 6.0% stage IV, 6.0% stage V CKD and 15.5% on dialysis. Patients taking ticagrelor had mean creatinine 3.5 ± 3.1, eGFR 31.5 ± 18.5, with 53.7% stage III, 13.7% stage IV, 10.4% stage V CKD and 22.1% on dialysis.

Patients taking ticagrelor had higher percentage of MI compared to patients taking clopidogrel (79.3% vs. 61.5%), similar severity of TIMI 0/1 (48.7% vs. 50.9%), LM stenosis (119% vs. 9.5%, *p* = 0.610), culprit artery territory (*p* = 0.095), LVEF (*p* = 0.594), treatment during admission with coronary intervention (*p* = 0.068) and coronary stenting (*p* = 0.378), concomitant disease: hypertension (*p* = 0.165) and dyslipidemia (*p* = 0.236). In general, there were substantial differences of the baseline characteristics between the two groups before propensity score adjustment (absolute STD > 0.2).

### 3.2. Primary Outcomes During Follow-Up

At 6 month follow-up, as shown in Table 2, patients taking ticagrelor had higher MI readmission compared to patients taking clopidogrel (hazard ratio [HR] 1.76, 95% confidence interval [CI] 1.12–2.77, *p* = 0.014), higher CV-related readmission (HR 2.16, 95% CI 1.24–3.77, *p* = 0.007), repeat revascularization (HR 3.34, 95% CI 1.66–6.68, *p* < 0.001) including PCI (HR 3.80, 95% CI 1.86–7.79, *p* < 0.001), and composite outcome (HR 1.97, 95% CI 1.15–3.40, *p* = 0.014).

At 1-year follow-up, patients taking ticagrelor had higher MI readmission compared to patients taking clopidogrel (HR 1.72, 95% CI 1.12–2.77, *p* = 0.014), higher CV-related readmission (HR 1.62, 95% CI 1.04–2.51, *p* = 0.032), and repeat revascularization (HR 2.63, 95% CI 1.56–4.43, *p* < 0.001) including PCI (HR 2.84, 95% CI 1.68–4.80, *p* < 0.001).

At 2-year follow-up, patients taking ticagrelor had higher MI readmission compared to patients taking clopidogrel (HR 1.59, 95% CI 1.12–2.28, *p* = 0.010), higher CV-related readmission (HR 1.72, 95% CI 1.12–2.65, *p* = 0.014), repeat revascularization (HR 2.24, 95% CI 1.36–3.68, *p* = 0.002) including PCI (HR 2.39, 95% CI 1.45–3.95, *p* = 0.001) and composite outcome (HR 1.63, 95% CI 1.06–2.48, *p* = 0.024).

**Figure 2:**
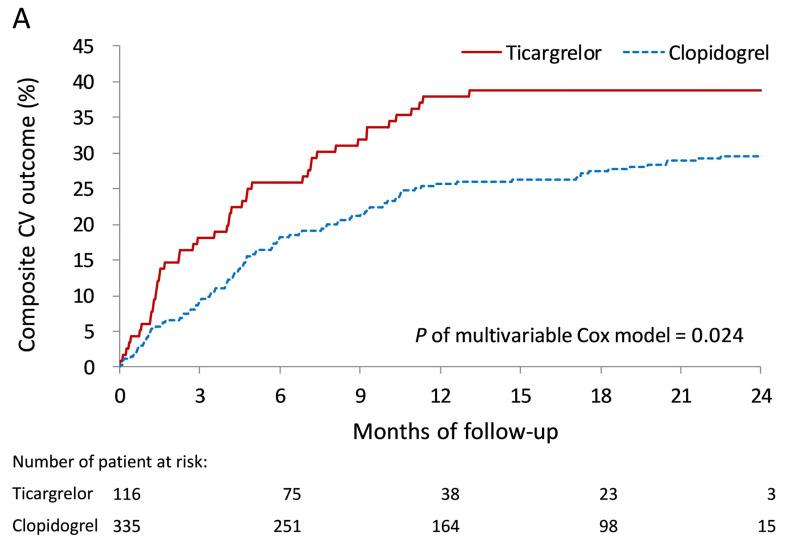

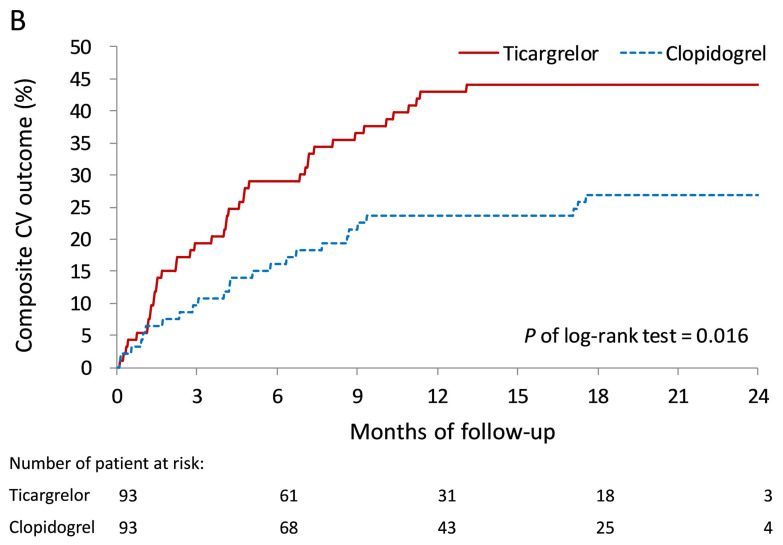
Cumulative event rates of composite CV outcome, including CV death, CV-related readmission, and repeated revascularization showing higher event rate with ticagrelor compared with clopidogrel, using expectation maximization (*p* = 0.0240) (**A**) and propensity score matching (*p* = 0.016) (**B**).

**Figure 3:**
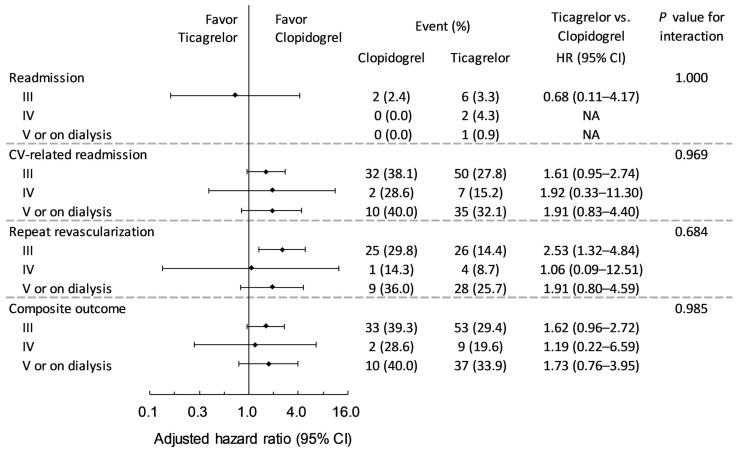
Forest plots of ticagrelor versus clopidogrel by myocardial infarction readmission (*p* = 1.000), CV-related readmission (*p* = 0.969), repeated revascularization (*p* = 0.684), and composite CV outcome (*p* = 0.985).

The cumulative event rates for the cumulative composite CV outcome, including CV death, CV-related readmission, and repeated revascularization, showing higher event rate with ticagrelor compared with clopidogrel, by propensity score adjustment (*p* = 0.0240) (Figure 2A) and propensity score matching (*p* = 0.016) (Figure 2B). Forest plots showed that there was no difference in the main study outcomes when comparing the use of ticagrelor versus clopidogrel by myocardial infarction readmission (*p* = 1.000), CV-related readmission (*p* = 0.969), repeated revascularization (*p* = 0.684), and composite CV outcome (*p* = 0.985) (Figure 3).

## 4. Discussion

In this retrospective cohort study focusing on type 2 diabetes mellitus patients with ACS and concomitant CKD, we found that DAPT with ticagrelor did not confer clinical advantages compared with clopidogrel. On the contrary, ticagrelor was associated with higher rates of CV-related readmission and repeated revascularization, resulting in an overall higher incidence of composite CV outcomes. These findings persisted after adjustment using both expectation maximization and propensity score matching, suggesting that the observed differences were not solely attributable to baseline imbalances.

Our results diverge from the pivotal PLATO trial, in which ticagrelor demonstrated superiority over clopidogrel in reducing all-cause mortality and MACE, including in patients with CKD subgroups [7,11]. In PLATO, the benefits of ticagrelor were consistent across invasive and non-invasive management strategies, with greater reductions in ischemic complications observed as renal function declined [20,21]. Pharmacodynamic studies also showed that ticagrelor provided more potent platelet inhibition than clopidogrel in CKD patients with ACS [22].

However, advanced CKD and dialysis patients were underrepresented in PLATO, limiting the generalizability of its findings to this population. By contrast, several subsequent studies conducted in East Asian populations have reported neutral or even unfavorable outcomes with ticagrelor. The PHILO trial in Japan showed numerically higher ischemic and bleeding events with ticagrelor compared with clopidogrel [12], and the TICAKOREA trial in Korea demonstrated no significant efficacy advantage but increased bleeding risk associated with ticagrelor [13]. Similarly, real-world studies from Taiwan and other East Asian countries suggested that ticagrelor may not offer superior ischemic protection in CKD or dialysis patients, while the risks of adverse events remained a concern [14,15]. A recent network meta-analysis further reinforced this observation by showing that the relative efficacy of ticagrelor in CKD was attenuated, whereas bleeding risk persisted [23].

Several mechanisms may account for these discrepancies. First, patient characteristics in our cohort differ substantially from those in PLATO, with our patients being older and having a greater burden of comorbidities. CKD itself alters platelet function, exacerbates endothelial dysfunction, and increases both thrombotic and bleeding risks, which may modify the net clinical effect of more potent platelet inhibition [22,24]. Prior studies have shown that despite greater platelet inhibition with ticagrelor, the expected ischemic benefit may be attenuated in CKD due to competing risks of bleeding and accelerated vascular disease progression [25]. In addition, uremia-related platelet dysfunction is an important pathophysiological feature of advanced CKD and may contribute to bleeding risk independently of antiplatelet drug choice. This mechanism could partly explain why ticagrelor did not translate into better net outcomes in our population, particularly in patients with late-stage CKD or dialysis.

Second, pharmacogenetic and ethnic differences may also play a role. Although East Asian patients have a higher prevalence of CYP2C19 loss-of-function alleles, which reduces clopidogrel responsiveness, clinical outcomes in East Asian trials and registries have not consistently shown ticagrelor to be superior [12,13]. This phenomenon, sometimes described as the “Asian paradox,” suggests that the net clinical balance in Asian populations may favor clopidogrel due to their generally lower ischemic risk and higher bleeding susceptibility [26,27].

Third, treatment selection and prescribing patterns in real-world practice may have contributed to our findings. In Taiwan, ticagrelor has often been preferentially prescribed to younger patients or those presenting with acute myocardial infarction, reflecting physicians’ perception of its greater potency. This channeling bias may inadvertently enrich the ticagrelor group with higher-risk patients who require more repeat revascularization and experience higher readmission rates, a phenomenon observed in another real-world cohort [15].

Finally, although ticagrelor is predominantly hepatically cleared and not directly renally excreted, CKD alters platelet turnover, oxidative stress, drug metabolism, and systemic inflammation [22,28]. These pathophysiological changes may attenuate the pharmacodynamic benefits of ticagrelor and explain why its biological potency does not necessarily translate into superior clinical outcomes in CKD populations. Notably, recent observational studies in advanced CKD and dialysis patients echo our findings, demonstrating neutral or even unfavorable ischemic outcomes with ticagrelor compared with clopidogrel [14,15]. Furthermore, renal hemodynamics may represent an additional prognostic factor in this population. Recent evidence suggests that a high renal resistive index is associated with adverse cardiorenal outcomes in CKD patients and may also influence drug responsiveness [29]. Although our registry did not capture such data, future studies incorporating renal hemodynamic markers may provide valuable insights into risk stratification and treatment optimization in ACS patients with CKD.

Our findings are also consistent with evidence from within Taiwan. A recent study by Tsai et al. compared ticagrelor with adjusted dose prasugrel in a real-world Taiwanese ACS population and found that prasugrel achieved a more favorable balance between ischemic protection and bleeding risk [16]. Although this study did not specifically focus on CKD, it underscores the importance of individualized antiplatelet strategies in Asian patients and suggests that more potent P2Y12 inhibitors may not uniformly provide net benefit in these populations. Together with our current findings, these results reinforce the notion that antiplatelet therapy decisions in CKD and East Asian patients should be guided by local data and patient-specific risk profiles rather than extrapolated solely from Western trial evidence.

This study has several limitations. First, the registry only contains data collected from the major medical facilities in Taiwan; therefore, not all patients with ACS in Taiwan were included in the analysis. As an observational analysis, residual confounding cannot be excluded despite the use of advanced statistical adjustments. A major limitation of this study is the potential for confounding by indication. In real-world practice, ticagrelor was often preferentially prescribed to younger or higher-risk patients, including those with more severe coronary disease. This channeling bias may have contributed to the observed differences in outcomes despite statistical adjustment. In addition, although some endpoints reached statistical significance, the confidence intervals were relatively wide, reflecting limited statistical precision and statistical power due to the modest sample size. These results should therefore be interpreted with caution. Second, another major limitation is the absence of bleeding outcomes in the registry. The well-recognized trade-off between ischemic protection and bleeding is central to evaluating the net clinical benefit of ticagrelor versus clopidogrel. Without bleeding data, the interpretation of our findings is incomplete and limited. Third, there were fewer patients taking ticagrelor compared to patients taking clopidogrel as part of DAPT in this cohort. The balancing effort by EM and propensity score adjustment in limited variables could not completely eliminate the difference between the two groups. Furthermore, information on adherence, duration of DAPT, drug intolerance, and adjunctive use of gastroprotective agents was unavailable. The pharmacogenomic data, such as CYP2C19 genotype, were not collected, which could have provided further mechanistic insight into clopidogrel responsiveness. Finally, as this study was conducted in the Taiwanese population, generalizability to other ethnicities should be interpreted with caution.

## 5. Conclusions

Among type 2 diabetes mellitus patients with ACS and CKD, ticagrelor use was associated with higher rates of CV-related readmission and revascularization compared with clopidogrel. However, these associations may be influenced by potential confounding factors, and no causal inferences can be drawn. While our findings are consistent with prior Asian and CKD-specific studies, they should be interpreted with caution. Dedicated randomized trials are needed to determine the optimal antiplatelet strategy in this high-risk population.

## Figures and Tables

**Table 1:** Baseline characteristics of the patients.

Variable	Missing Value (no.)	Ticagrelor (*n* = 116)	Clopidogrel (*n* = 335)	*p* Value	STD
Male sex, n (%)	0	78 (67.2)	223 (66.6)	1.000	0.01
Age (years)	0	66.6 ± 11.2	68.7 ± 10.5	0.062	−0.19
Body mass index (kg/m^2^)	56	25.9 ± 3.5	26.0 ± 4.2	0.712	−0.03
Waist circumference (cm)	378	91.8 ± 6.4	93.1 ± 8.2	0.130	−0.18
Creatinine (mg/dL)	8	3.0 ± 2.9	3.5 ± 3.1	0.108	−0.17
eGFR (mL/min/1.73 m^2^)	8	36.9 ± 18.2	31.5 ± 18.5	0.006	0.29
CKD stage, n (%)	0			0.004	
III		84 (72.4)	180 (53.7)		0.39
IV		7 (6.0)	46 (13.7)		−0.26
V		7 (6.0)	35 (10.4)		−0.16
On dialysis		18 (15.5)	74 (22.1)		−0.17
DM duration (years)	138	11.2 ± 7.0	12.4 ± 7.3	0.115	−0.17
Etiology of ACS, n (%)	0			0.001	
Atypical chest pain		13 (11.2)	56 (16.7)		−0.16
Unstable angina		11 (9.5)	73 (21.8)		−0.34
Myocardial Infarction		92 (79.3)	206 (61.5)		0.40
Culprit lesion	0			<0.001	
50–70%		6 (1.8)	2 (1.7)		0.01
70–90%		124 (37.0)	11 (9.5)		0.69
≥90%		205 (61.2)	103 (88.8)		−0.67
TIMI flow	0			0.684	
Occluded (TIMI 0/1)		163 (48.7)	59 (50.9)		−0.04
Slow (TIMI 2)		36 (10.7)	8 (6.9)		0.13
Normal (TIMI 3)		76 (22.7)	26 (22.4)		0.01
Unknown or Missing		60 (17.9)	23 (19.8)		−0.05
LM stenosis	0	40 (11.9)	11 (9.5)	0.610	
Number of vessels	0			0.105	
0 (including normal)		(0.0)	1 (0.9)		
I		100 (29.9)	51 (44.0)		−0.30
II		91 (27.2)	23 (19.8)		0.18
III		104 (31.0)	30 (25.9)		0.11
LM		1 (0.3)	(0.0)		0.08
LM + I		4 (1.2)	1 (0.9)		0.03
LM + II		8 (2.4)	3 (2.6)		−0.01
LM + III		27 (8.1)	7 (6.0)		0.08
Culprit artery territory	0			0.095	
LM		9 (2.7)	3 (2.6)		0.01
LAD		147 (43.9)	37 (31.9)		0.25
LCx		56 (16.7)	25 (21.6)		−0.12
RCA		113 (33.7)	50 (43.1)		−0.19
None or missing		10 (3.0)	1 (0.9)		0.15
LVEF	0			0.594	
Normal		134 (40.0)	49 (42.2)		−0.04
40–50%		77 (23.0)	21 (18.1)		0.12
30–40%		33 (9.9)	16 (13.8)		−0.12
<30%		22 (6.6)	9 (7.8)		−0.05
Missing		69 (20.6)	21 (18.1)		0.06
Treatment during the admission					
Coronary intervention	0	110 (94.8)	298 (89.0)	0.068	0.21
Coronary stenting	0	107 (92.2)	297 (88.7)	0.378	0.12
Concomitant disease, n (%)					
Hypertension	0	99 (85.3)	303 (90.4)	0.165	−0.16
Dyslipidemia	0	58 (50.0)	189 (56.4)	0.236	−0.13
History of event, n (%)					
Known CAD	0	48 (41.4)	159 (47.5)	0.281	−0.12
Old myocardial infarction	0	20 (17.2)	77 (23.0)	0.238	−0.15
Previous PCI	0	33 (28.4)	118 (35.2)	0.210	−0.15
Previous CABG	0	2 (1.7)	31 (9.3)	0.006	−0.34
Atrial fibrillation	0	2 (1.7)	18 (5.4)	0.120	−0.20
Heart failure	0	10 (8.6)	42 (12.5)	0.313	−0.13
COPD	0	6 (5.2)	14 (4.2)	0.610	0.05
Peripheral artery disease	0	3 (2.6)	29 (8.7)	0.034	−0.27
Cancer	0	1 (0.9)	17 (5.1)	0.053	−0.25
Ischemic stroke	0	4 (3.4)	38 (11.3)	0.009	−0.31
Hemorrhagic stroke	0	6 (5.2)	8 (2.4)	0.209	0.15
Lipid profile					
Troponin I (ng/mL)	38	1.18 [0.17, 9.08]	1.00 [0.09, 13.16]	0.593	NA
LDL-C (mg/dL)	79	96.4 ± 29.7	92.9 ± 34.6	0.326	0.11
Total cholesterol (mg/dL)	60	163.5 ± 38.7	158.0 ± 42.0	0.216	0.14
HDL-C (mg/dL)	115	38.6 ± 10.5	39.1 ± 10.6	0.634	−0.05
Triglyceride (mg/dL)	66	165.4 ± 123.7	154.7 ± 104.2	0.365	0.09
NT-proBNP (mg/dL)	381	9191 [3660, 16,460]	14,620 [7552, 21,796]	<0.001	NA
HbA1C (%)	183	8.1 ± 1.5	7.8 ± 1.2	0.013	0.22
Fasting glucose (mg/dL)	136	214.8 ± 75.4	200.3 ± 83.6	0.099	0.18
Hemoglobin (mg/dL)	8	12.4 ± 2.1	11.8 ± 2.5	0.020	0.26
Smoking, n (%)	0	27 (23.3)	79 (23.6)	1.000	−0.01
Lowering glucose drug, n (%)					
Insulin	0	52 (44.8)	123 (36.7)	0.124	0.17
Sulfonylurea	0	40 (34.5)	106 (31.6)	0.567	0.06
Mitiglinide	0	12 (10.3)	47 (14.0)	0.342	−0.11
Metformin	0	34 (29.3)	75 (22.4)	0.166	0.16
Thiazolidinedione	0	1 (0.9)	6 (1.8)	0.683	−0.08
Acarbose	0	11 (9.5)	24 (7.2)	0.424	0.08
DPP4 inhibitor	0	49 (42.2)	130 (38.8)	0.512	0.07
Non-DM medication, n (%)					
Aspirin	0	109 (94.0)	294 (87.8)	0.079	0.22
Anticoagulant	0	2 (1.7)	10 (3.0)	0.739	−0.09
GP IIb/IIIa Inhibitor	0	7 (6.0)	9 (2.7)	0.140	0.16
Heparin or LMWH	0	76 (65.5)	223 (66.6)	0.909	−0.02
ACEI or ARB	0	76 (65.5)	192 (57.3)	0.126	0.17
Beta-blocker	0	86 (74.1)	222 (66.3)	0.133	0.17
Statin	0	86 (74.1)	257 (76.7)	0.614	−0.06
Calcium channel blocker	0	25 (21.6)	139 (41.5)	<0.001	−0.44
Digoxin	0	2 (1.7)	11 (3.3)	0.530	−0.10
Diuretic	0	37 (31.9)	155 (46.3)	0.009	−0.30
IV Inotropic agent	0	5 (4.3)	35 (10.4)	0.057	−0.24
Nitrate	0	49 (42.2)	156 (46.6)	0.450	−0.09
Antiarrhythmic drug	0	11 (9.5)	38 (11.3)	0.729	−0.06
Proton pump inhibitor	0	42 (36.2)	125 (37.3)	0.911	−0.02
Follow up duration (year)	0	1.3 ± 0.7	1.5 ± 0.6	0.027	−0.31

Abbreviation: STD, standardized difference; eGFR, estimated glomerular filtration rate; CKD, chronic kidney disease; DM, diabetes mellitus; ACS, acute coronary syndrome; TIMI, Thrombolysis In Myocardial Infarction; LM, left main; LAD, left anterior descending; LCx, circumflex; RCA, right coronary artery; LVEF, left ventricular ejection fraction; CAD, coronary artery disease; PCI, percutaneous coronary intervention; CABG, coronary artery bypass graft; COPD, chronic obstructive pulmonary disease; LDL-C, low-density lipoprotein cholesterol; NA, not applicable; HDL-C, high-density lipoprotein cholesterol; GP, glycoprotein; LMWH, low molecular weight heparin, ACEI, angiotensin converting enzyme inhibitor, ARB, angiotensin II receptor blocker; Data were given as frequency (%), mean ± standard deviation or median [25th and 75th percentiles].

**Table 2:** Follow up outcomes between the study cohorts.

	Number of Event (%)		Unadjusted Analysis		Adjusted Analysis *	
Outcome	Ticagrelor (*n* = 116)	Clopidogrel (*n* = 335)	HR (95% CI) of Ticagrelor	*p*	HR (95% CI) of Ticagrelor	*p*
6 month follow-up						
Death	7 (6.0)	14 (4.2)	1.56 (0.63–3.87)	0.334	2.35 (0.80–6.87)	0.118
CV death	1 (0.9)	4 (1.2)	0.79 (0.09–7.03)	0.829	0.79 (0.06–9.60)	0.850
Readmission	39 (33.6)	87 (26.0)	1.46 (1.00–2.13)	0.049	1.76 (1.12–2.77)	0.014
CV-related readmission	28 (24.1)	48 (14.3)	1.95 (1.22–3.10)	0.005	2.16 (1.24–3.77)	0.007
Repeat revascularization	21 (18.1)	24 (7.2)	2.87 (1.60–5.16)	<0.001	3.34 (1.66–6.68)	0.001
PCI	20 (17.2)	22 (6.6)	3.03 (1.65–5.54)	<0.001	3.80 (1.86–7.79)	<0.001
CABG	2 (1.7)	2 (0.6)	3.07 (0.43–21.82)	0.261	2.16 (0.21–22.07)	0.516
Composite outcome #	29 (25.0)	52 (15.5)	1.86 (1.18–2.93)	0.008	1.97 (1.15–3.40)	0.014
1 year follow-up						
Death	9 (7.8)	30 (9.0)	0.94 (0.45–1.98)	0.870	1.26 (0.53–2.98)	0.603
CV death	2 (1.7)	8 (2.4)	0.78 (0.17–3.68)	0.755	0.46 (0.08–2.61)	0.383
Readmission	57 (49.1)	140 (41.8)	1.34 (0.99–1.83)	0.059	1.72 (1.19–2.49)	0.004
CV-related readmission	43 (37.1)	78 (23.3)	1.59 (1.09–2.32)	0.015	1.62 (1.04–2.51)	0.032
Repeat revascularization	35 (30.2)	47 (14.0)	2.53 (1.63–3.91)	<0.001	2.63 (1.56–4.43)	<0.001
PCI	35 (30.2)	45 (13.4)	2.67 (1.71–4.15)	<0.001	2.84 (1.68–4.80)	<0.001
CABG	2 (1.7)	2 (0.6)	3.07 (0.43–21.82)	0.261	2.16 (0.21–22.07)	0.516
Composite outcome #	44 (37.9)	85 (25.4)	1.49 (1.03–2.15)	0.034	1.49 (0.97–2.29)	0.069
2 year follow-up						
Death	11 (9.5)	41 (12.2)	0.85 (0.44–1.66)	0.638	1.29 (0.60–2.80)	0.514
CV death	2 (1.7)	9 (2.7)	0.71 (0.15–3.28)	0.659	0.48 (0.09–2.66)	0.403
Readmission	60 (51.7)	161 (48.1)	1.25 (0.93–1.68)	0.139	1.59 (1.12–2.28)	0.010
CV-related readmission	44 (37.9)	92 (27.5)	1.67 (1.16–2.39)	0.005	1.72 (1.12–2.65)	0.014
Repeat revascularization	35 (30.2)	58 (17.3)	2.09 (1.37–3.18)	0.001	2.24 (1.36–3.68)	0.002
PCI	35 (30.2)	56 (16.7)	2.19 (1.43–3.33)	<0.001	2.39 (1.45–3.95)	0.001
CABG	2 (1.7)	2 (0.6)	3.07 (0.43–21.82)	0.261	2.16 (0.21–22.07)	0.516
Composite outcome #	45 (38.8)	99 (29.6)	1.59 (1.12–2.27)	0.010	1.63 (1.06–2.48)	0.024

Abbreviation: CI, confidence interval; HR, hazard ratio; CV, cardiovascular; PCI, percutaneous coronary intervention; CABG, coronary artery bypass graft; * Adjusted for propensity score; # Anyone of CV death, repeated revascularization or CV-related readmission.

## Data Availability

The article includes the original contributions presented in this study. Further inquiries can be directed to the corresponding authors.
